# Phytocompounds targeting epigenetic modulations: an assessment in cancer

**DOI:** 10.3389/fphar.2023.1273993

**Published:** 2024-03-26

**Authors:** Aqsa Khan, Asifa Khan, Mohammad Aasif Khan, Zoya Malik, Sheersh Massey, Rabea Parveen, Saad Mustafa, Anas Shamsi, Syed A. Husain

**Affiliations:** ^1^ Department of Bioscience, Faculty of Natural Sciences, Jamia Millia Islamia (A Central University), New Delhi, India; ^2^ Department of Radiation Oncology, The University of Texas Health Science Centre at San Antonio, San Antonio, TX, United States; ^3^ Center for Medical and Bio-Allied Health Sciences Research, Ajman University, Ajman, United Arab Emirates

**Keywords:** cancer, cancer epigenetics, DNMTs, HDACs, miRNAs, phytocompounds, bioactive compounds, cancer prevention

## Abstract

For centuries, plants have been serving as sources of potential therapeutic agents. In recent years, there has been a growing interest in investigating the effects of plant-derived compounds on epigenetic processes, a novel and captivating Frontier in the field of epigenetics research. Epigenetic changes encompass modifications to DNA, histones, and microRNAs that can influence gene expression. Aberrant epigenetic changes can perturb key cellular processes, including cell cycle control, intercellular communication, DNA repair, inflammation, stress response, and apoptosis. Such disruptions can contribute to cancer development by altering the expression of genes involved in tumorigenesis. However, these modifications are reversible, offering a unique avenue for therapeutic intervention. Plant secondary compounds, including terpenes, phenolics, terpenoids, and sulfur-containing compounds are widely found in grains, vegetables, spices, fruits, and medicinal plants. Numerous plant-derived compounds have demonstrated the potential to target these abnormal epigenetic modifications, including apigenin (histone acetylation), berberine (DNA methylation), curcumin (histone acetylation and epi-miRs), genistein (histone acetylation and DNA methylation), lycopene (epi-miRs), quercetin (DNA methylation and epi-miRs), *etc.* This comprehensive review highlights these abnormal epigenetic alterations and discusses the promising efficacy of plant-derived compounds in mitigating these deleterious epigenetic signatures in human cancer. Furthermore, it addresses ongoing clinical investigations to evaluate the therapeutic potential of these phytocompounds in cancer treatment, along with their limitations and challenges.

## 1 Introduction

Despite modern advances and therapeutics, cancer remains one of the most dreaded diseases globally. According to the GLOBOCAN cancer statistics 2020, female breast cancer incidence superseded lung cancer globally. Around 19.3 million new cancer cases have been reported worldwide, with 10 million cancer deaths. With a record of 2.3 million new diagnoses, female breast cancer has surpassed lung cancer as the most frequently diagnosed cancer, followed by colorectal (10.0%), prostate (7.3%), and abdominal (5.6%) cancers respectively. In 2040, the global cancer incidence is predicted to increase to 28.4 million cases, increasing 47% from 2020 (Sung et al., 2021). This creates an alarming situation towards the increasing number of cancer cases and calls for better and safer treatment options.

Cancer is a multifaceted disease with genetic mutations and abnormal epigenetic modifications (Baylin et al., 2001). According to Conrad Hal Waddington, epigenetics refers to heritable and reversible modifications in gene expression or changes in a chromosome without any alterations in DNA sequence (Henikoff and Matzke, 1997). The epigenetic regulation includes DNA methylation, posttranslational histone protein modifications such as acetylation, methylation, and phosphorylation, and microRNAs (noncoding RNA) that can inhibit translation or degrade mRNAs by modulating gene expression (Dehan et al., 2009). Epigenetic control plays a crucial role during early embryonic development, such as X-inactivation in females, genomic imprinting, development, and differentiation like the formation of long-term memory and behavior (Payer and Lee, 2008; Lubin et al., 2011). In addition to this, research studies suggest that epigenetic DNA modifications on uniparental disomy also serves as an underlying mechanism for developmental disorders, neurological diseases and tumorigenesis (Zoghbi and Beaudet, 2016; Tuna et al., 2009). A study on colorectal cancer showed several tumor suppressor genes (*VCAN*, *FLT4*, *SFRP1* and *GAS7*) in the uniparental disomy and polysomy regions displaying elevated levels of DNA methylation (Torabi et al., 2015).

Deregulation of epigenetic modifications has critical implications on human health, such as cancer, metabolic syndrome, and neurodegenerative disorders like Alzheimer’s disease, Huntington’s disease, and amyotrophic lateral sclerosis (Berson et al., 2018; Smith and Ryckman, 2015). Recent studies highlighted that maternal behavioral patterns, diet choice, and exposure to other intrinsic or extrinsic factors have dramatically altered gene expression patterns and are implicated with epigenetic mechanisms (Chung and Herceg, 2020; Safi-Stibler and Gabory, 2020).

Since epigenetic modifications are reversible, a significant number of studies are now focused on the identification and development of pharmaceuticals that target these modulations (Singh et al., 2016; Liu Z. et al., 2019). In recent years, Food and Drug Administration, United States, approved several small synthetic molecule inhibitors that target distinct epigenetic modulations to treat several solid tumors and hematological malignancies. These clinically approved conventional drugs include Azacytidine and Decitabine (DNA methyltransferases (DNMT) inhibitors). Four histone deacetylases inhibitors (HDACi) are approved for the treatment of lymphomas, including vorinostat (SAHA), romidepsin (FK-228), belinostat (PXD-101), and tucidinostat (chidamide) (previous conditional approvals for panobinostat [LBH-589] for multiple myeloma [in combination with bortezomib] and romidepsin for peripheral T-cell lymphoma [PTCL] were recently withdrawn by the FDA) (Liu Z. et al., 2019; Xiao et al., 2023). On the contrary, several studies have found phytocompounds as potential regulators that can reverse these aberrant epigenetic modifications that induce tumor progression and eventually lead to cancer (Aggarwal et al., 2015; Montgomery and Srinivasan, 2019).

Drug development using naturally derived bioactive compounds has drawn a lot of interest in recent years as they are safe and economical. In contrast, present-day chemotherapeutic drugs are not only expensive but also toxic. One significant side effect of conventional medications is their ability to target normal cells in the body undergoing rapid proliferation, such as bone marrow cells, along with proliferating tumor cells. However, phytocompounds are nontoxic to normal cells and hence better tolerated (Singh et al., 2016). Phytocompounds are bioactive compounds, obtained from a wide variety of herbs, spices, vegetables, and fruits (Table 1), that possess anti-inflammatory, antioxidant, antimicrobial, anticancer, and anti-diabetic properties (Verma, 2016; Altaf et al., 2018; Gonelimali et al., 2018; Salehi et al., 2019). Furthermore, phytocompounds can target multiple cell cycle proteins, transcription factors, cell adhesion molecules, protein kinases, and anti-apoptotic factors (Aggarwal and Shishodia, 2006; Kundu and Surh, 2009; Bailon-Moscoso et al., 2017; Malik et al., 2021). Besides this, plant-based compounds possess the ability to target deregulated metabolic proteins and pathways in cancer, such as glycolysis, pentose phosphate pathway, lipid metabolism, amino acid metabolism, *etc.* (Pani et al., 2020; Khan et al., 2021a; Khan et al., 2021b). These multipronged functions of phytocompounds, targeted at various cellular pathways implicated epigenetically in cancer, can be a promising solution in the treatment of this deadliest disease.

**TABLE 1:** List of phytocompounds, their source and types.

Dietary phytocompound	Source	Type	Structure
Allicin	Allium sativum (Garlic)	Organosulfur compound	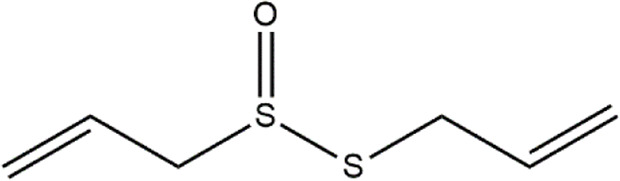
Allyl isothiocyanate	Garlic, broccoli, wasabi	Organosulfur compound	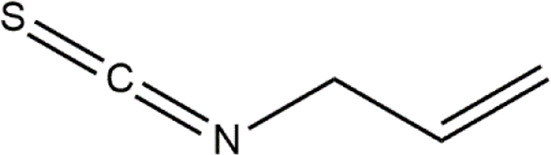
Apigenin	Fruits, vegetables, herbs (Parsley and chamomile)	Polyphenol (Flavonoid)	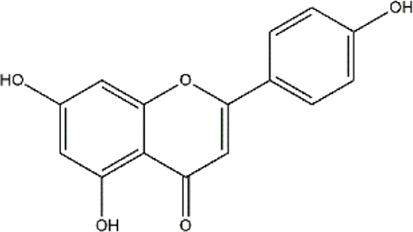
Berberine	*Berberis vulgaris* (barberry), *Berberis aristata* (tree turmeric)	Alkaloid	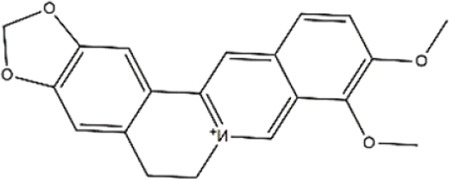
Celastrol	*Tripterygium wilfordii, Tripterygium regelii*	Triterpenoid	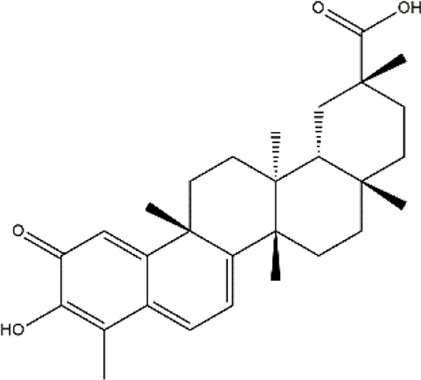
Cucurbitacin B	Cucumis sativus (Cucumber)	Triterpenoid	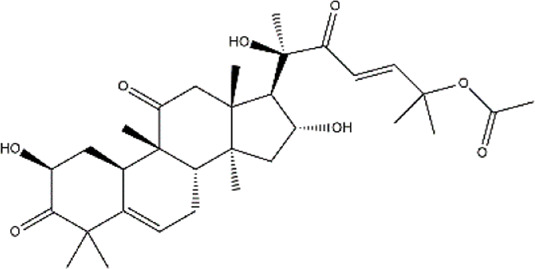
Curcumin	*Curcumin longa* (Turmeric)	Polyphenol (Flavonoid)	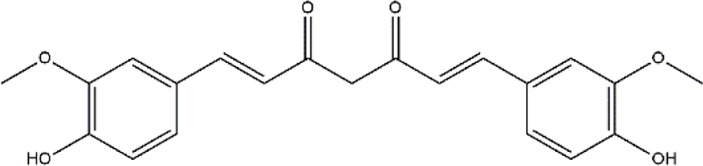
Daidzein	Soya beans and legumes	Polyphenol (Isoflavone)	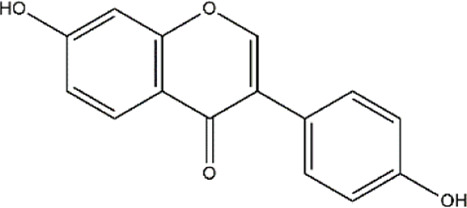
Diallyl disulfide	*Allium* genus	Organosulfur compound	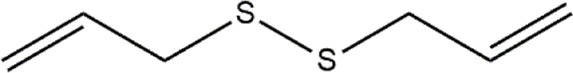
Demethylzeylasteral	*Tripterygium wilfordii* (three wingnut root)	Triterpenoid	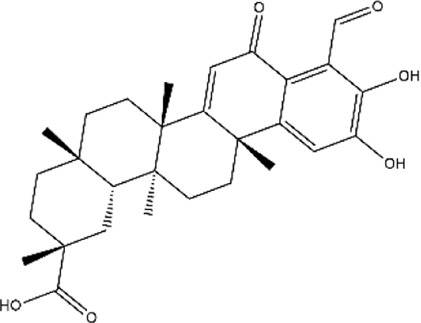
3,3′- Diindolylmethane	Cruciferous vegetables	Glucosinolates	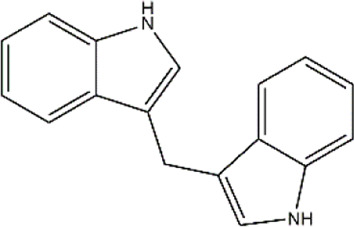
Ellagitannin	*Punica granatum* (Pomegranate) berries	Polyphenol	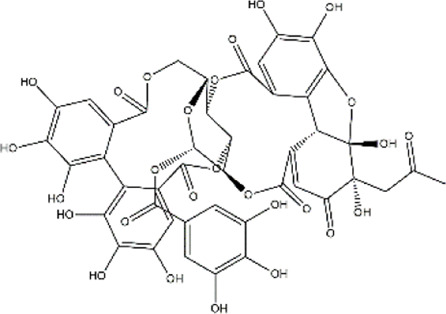
Epigallocatechin 3-gallate	*Camellia sinensis* (Green Tea)	Polyphenol (Catechol)	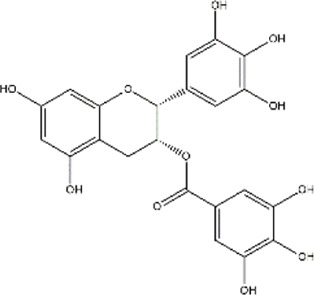
Garcinol	*Garcinia indica* (kokum)	Benzophenone	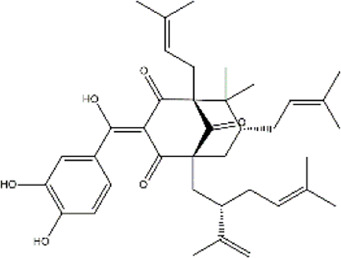
Genistein	*Glycine* max (soya beans)	Polyphenol (Isoflavone)	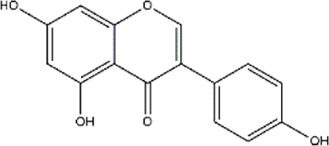
Ginsenoside Rh2	Panax ginseng (Korean ginseng)	Ginsenoside	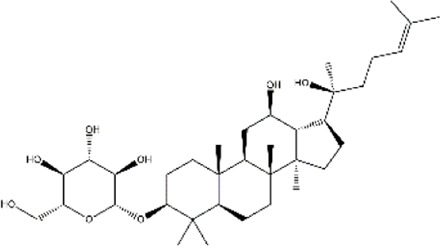
Glabridin	*Glycyrrhiza glabra* (Licorice)	Polyphenol (Isoflavone)	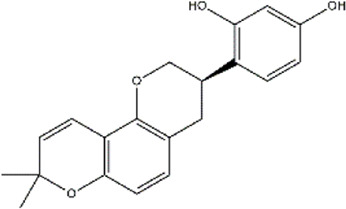
Gossypol	*Gossypium hirsutum* (cotton plant)	Phenol	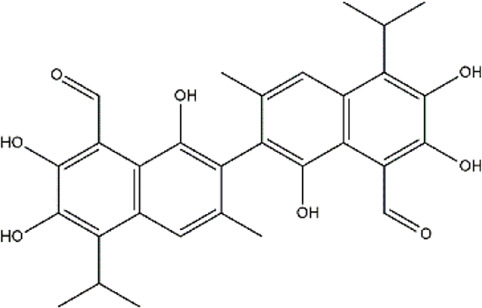
Icariin	*Herba epimedii* (Yin-yang-huo)	Polyphenol (Flavonoid)	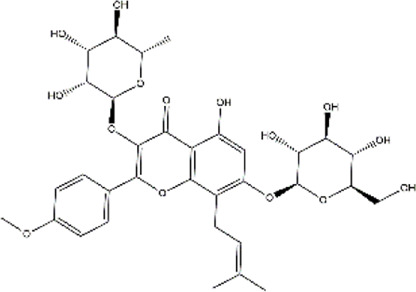
Indole-3-carbinol	Cruciferous vegetables	Glucosinolates	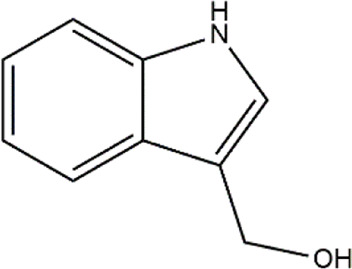
Kaempferol	Fruits, vegetables and herbs	Polyphenol (Flavonoid)	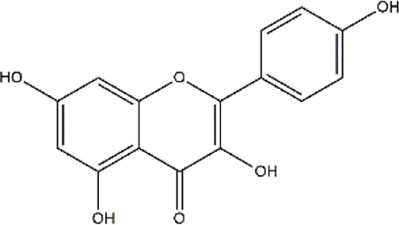
Luteolin	Parsley, broccoli, carrots, peppers, cabbage	Polyphenol (Flavonoid)	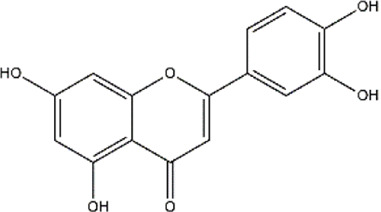
Lycopene	Tomato, papaya, watermelon	Carotenoid	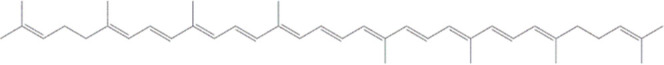
Methyl jasmonate	*Jasminum grandiflorum* (Spanish Jasmine)	Methyl ester	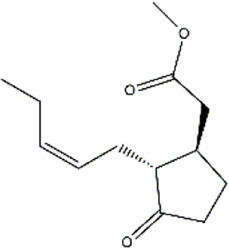
Parthenolide	*Tanacetum parthenium* (Feverfew)	Sesquiterpene lactone	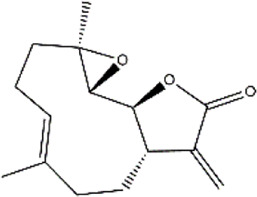
Phenethyl isothiocyanate	Cruciferous vegetables	Isothiocyanate	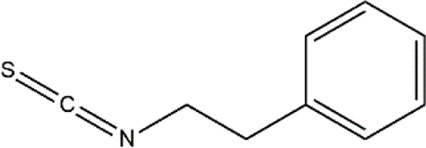
Piceatannol	Red wine, grapes, passion fruit	Stilbene	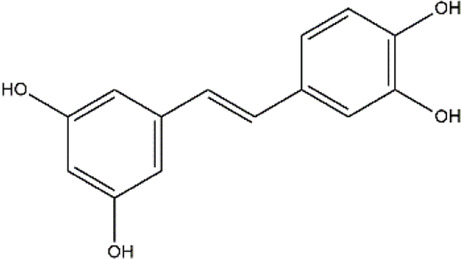
Quercetin	Fruits, vegetables, tea, red wine, nuts, propolis	Polyphenol (Flavonoid)	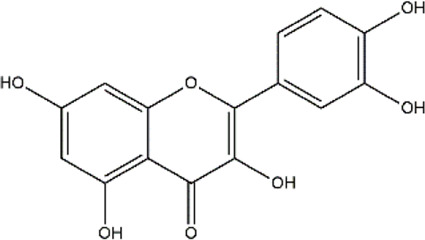
Resveratrol	Grapes, berries	Polyphenol	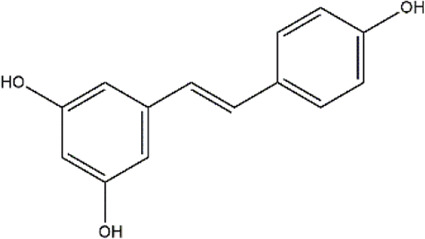
Rosmarinic acid	*Rosmarinus officinalis* (rosemary)	Polyphenol	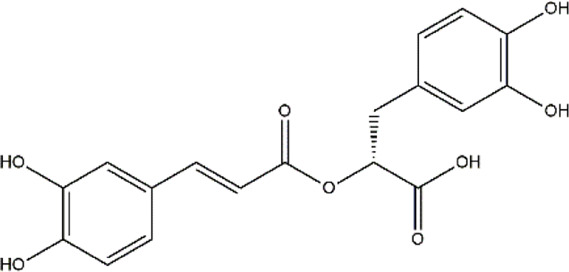
Shikonin	*Lithospermum erythrorhizon* (purple gromwell)	Naphthoquinone	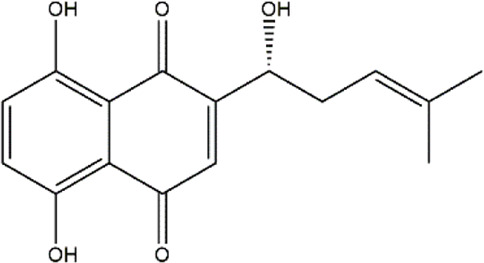
Sulforaphane	Cruciferous vegetables	Isothiocyanate	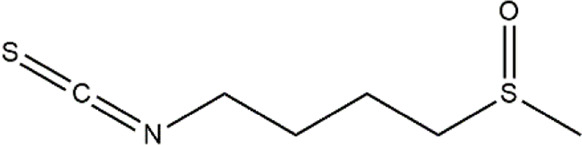
Thymoquinone	*Monarda fistulosa* (wild bergamot)*, Nigella sativa* (black cumin)	Terpenoid	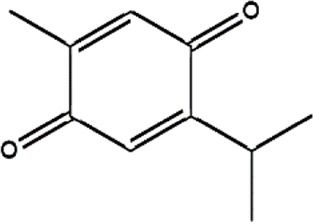
Triptolide	*Tripterygium wilfordii* (Three wingnut Root)	Terpenoid	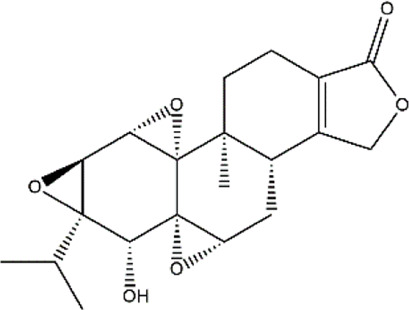
Ursolic acid	*Oldenlandia diffusa* (Snake-needle grass)*, Radix actinidiae* (kiwi root)	Terpenoid	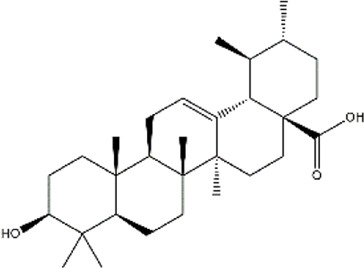
Withaferin A	*Withania somnifera* (ashwagandha)	Lactone	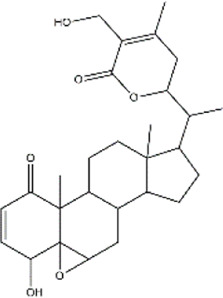

**FIGURE 1:**
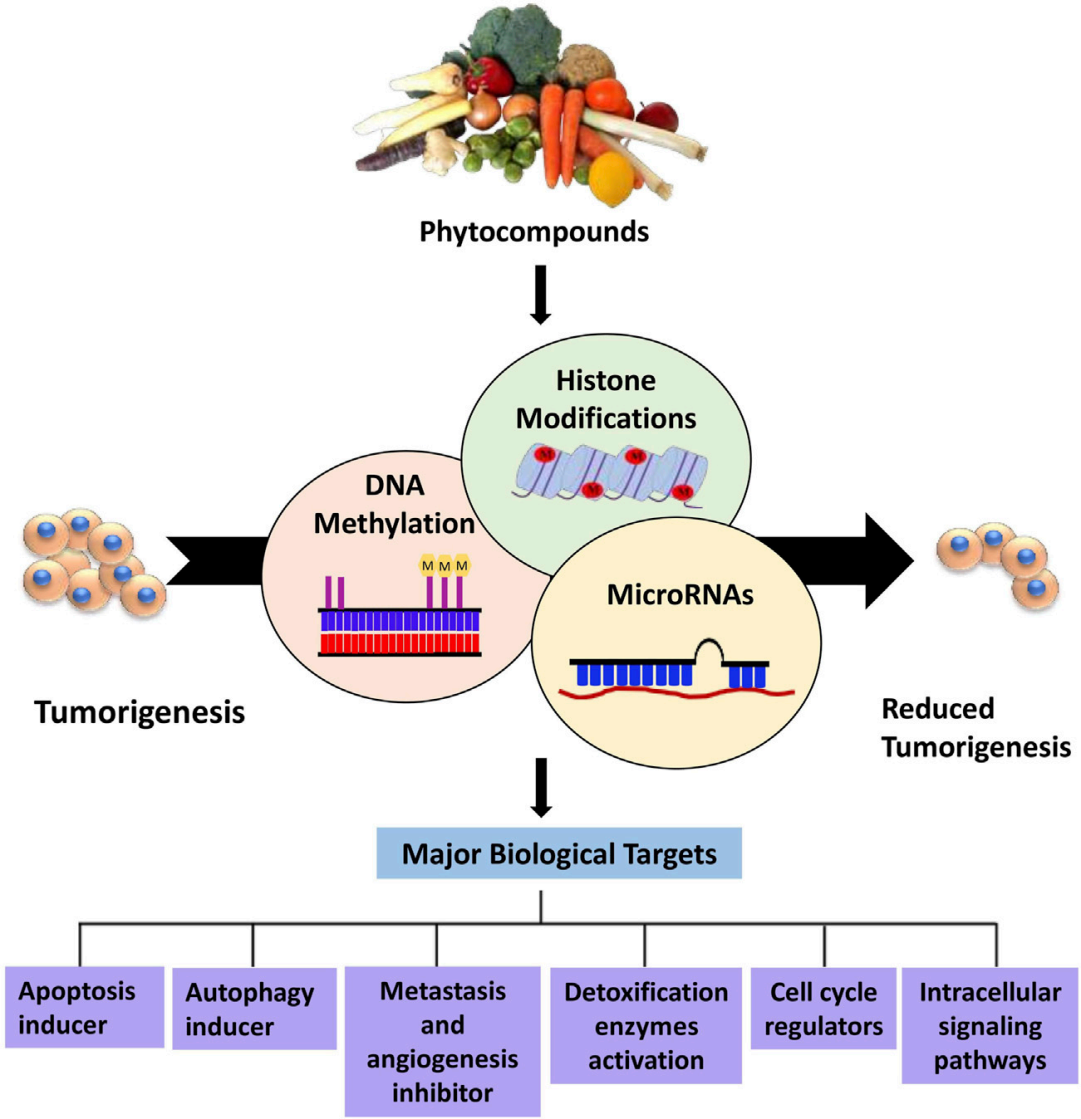
Overview of phytocompounds regulating major epigenetic modifications that involve regulation of key biological targets to reduce tumorigenesis.

This review highlights the prospective use of distinct phytocompounds to counteract epigenetic abnormalities that promote tumor development and progression in humans as well as their potential in anticancer therapeutics as summarized in Figure 1. Therefore, a comprehensive explanation of the most studied dietary compounds that critically modulate the epigenetic landscape in human cancer along with the latest developments, is provided extensively. In addition, list of all the phytocompounds that are discussed in this review and that are known to control epigenetic alterations are listed below in Table 1, along with their classification and sources.

## 2 Method

**FIGURE 2:**
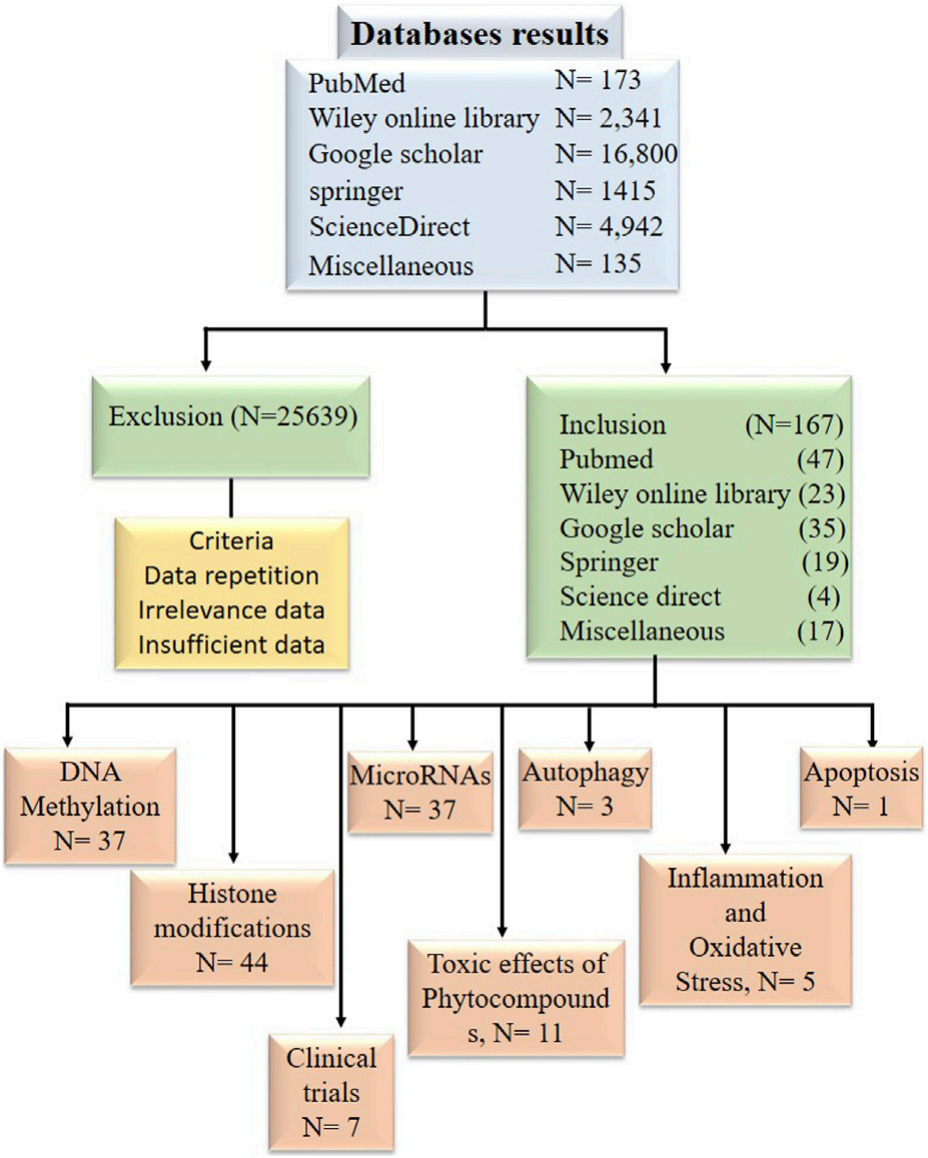
Diagram in block form illustrating the inclusion and exclusion of research publications in data tabulation.

Research articles, reviews, and abstracts were pulled for a literature survey from a variety of databases, including PubMed, Google Scholar, Springer, Wiley Online Library, ScienceDirect, *etc.*, From the year 1998 and to 2022. Information from research articles, review articles, abstracts were used for this review. The literature search was conducted against the terminology “Bioactive compounds in cancer epigenetics” while other terminologies such as phytocompounds targeting DNA methylation, histone modification and epi-miRNAs were also included in this search as illustrated in Figure 2. Data was processed to find out general information about phytocompounds, with a strong focus on how they affect epigenetics modifications. Inclusion criteria was based on the topics that include phytocompounds targeting epigenetic modifications (DNA methylation, Histone modifications and miRNAs) in cancer. While the exclusion criteria, was based on studies demonstrating information duplication, such as reviews, material available in languages other than English, information unrelated to the subjects included in this analysis, insufficient information, and data older than 1998.

### 2.1 DNA methylation

DNA methylation is responsible for controlling gene expression and interacting with the nucleosomes that control DNA packaging, and it can influence entire DNA domains. It is a chemical, biological modification where cytosine residues are methylated at a 5′position. This modification mainly occurs at cytosine residues present in GC dinucleotide-rich regions clustered together to form the CpG islands spanned the 5′end region of several genes (Issa and Kantarjian, 2009).

### 2.2 DNA methyltransferases

**FIGURE 3:**
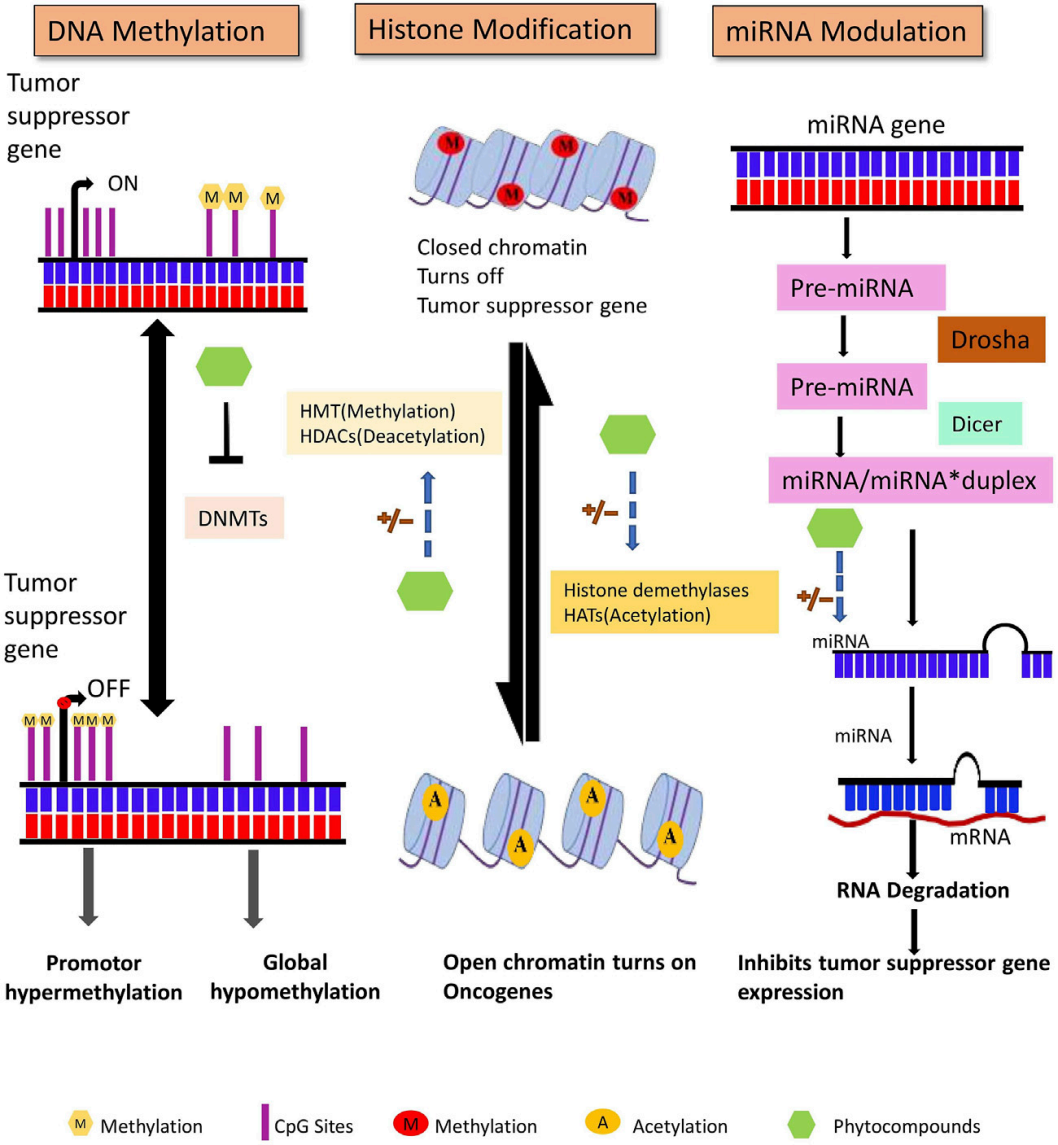
DNA methylation, posttranslational histone modifications, and microRNAs are all major epigenetic processes that regulate gene expression. These pathways are found to be dysregulated in cancer. Phytocompounds have shown to modulate abnormal epigenetic modifications.

The human genome contains approximately 28 million CpG sites. Under normal circumstances, most CpG islands, especially within the promoter region, are unmethylated except in the case of X-inactivation, genome imprinting, and repression of transposons. DNA methylation occurs during the early embryonic stages by a set of distinct enzymes called DNA methyltransferases (DNMTs) (Bird, 2002). DNA methyltransferases catalyze the formation of 5-methyl cytosine that involves the transfer of a methyl group from S-adenosyl-L- methionine (SAM) to the 5′position of the cytosine residue in CG dinucleotide (Santi et al., 1983). DNMT1, DNMT3A, and DNMT3B are the three major DNMTs involved in DNA methylation in mammals. Maintenance of methylation patterns during replication is one of the critical functions of DNMT1. During replication, DNMT1 adds a methyl group to hemi methylated CpG dinucleotides in the daughter strand as it shows 5–30 times greater affinity for hemi methylated substrates. In contrast, DNMT3A and DNMT3B are *de novo* methyltransferases exclusively involved in the methylation of previously unmethylated DNA sequences (Okano et al., 1999; Bestor, 2000). To ensure methylation, DNMTs must have access to the DNA that can be gained via perturbation of chromatin structure by specific chromatin remodeling proteins (As shown in Figure 3) (Baylin et al., 2001).

In cancer, hypermethylation of CpG islands in the promoter sequence of some genes results in gene silencing by suppressing the transcriptional activity of tumor suppressor genes (Dehan et al., 2009). For instance, promoter hypermethylation of CpG sites of tumor suppressor genes like *RARβ* and *RASSF1A* can induce breast cancer development (Van Hoesel et al., 2013). Certain studies of malignant and tumor cells have shown hypomethylation of DNA sequences at CpG sites that can cause conformational and functional alterations in chromosomes (Gama-Sosa et al., 1983). In cancer there are several natural compounds known to influence the DNA methylation as listed in Table 2 in most of the cases by a single publication.

**TABLE 2:** List of phytocompounds modulating DNA methylation.

Dietary phytocompound	Mechanism	Genes targeted	Cancer type (cancer cell line)	References
Apigenin	DNMT inhibitor, Promoter demethylation	*Nrf2*	Skin (JB6 P + -mouse cell line)	Paredes-Gonzalez et al. (2014)
Berberine	DNMT inhibitor	*p53*	Hepatocellular carcinoma (Hep3B)	Kim et al. (2020)
Cucurbitacin B	Promoter hypermethylation	*c-Myc, cyclin D1, survivin*	Breast (MDA-MB-231, MCF-7)	Dittharot et al. (2019)
Curcumin	DNMT inhibitor, promoter demethylation	*BRCA1, SNCG, TET1*	Breast (HCC-38, UACC-3199, T47D)	Al-Yousef et al. (2020)
		*Neurog-1*	Prostate (LNCaP)	Shu et al. (2011)
	Promoter hypomethylation	*RARβ*	Lung (A549, H460)	Jiang et al. (2015)
Daidzein	DNMT inhibitor, Promoter demethylation	*BRCA1, EPHB2 and GSTP1*	Prostate (DU-145, PC-3)	Adjakly et al. (2011)
Epigallocatechin 3-gallate (EGCG)	DNMT inhibitor	*p16INK4a, RARβ, MGMT, hMLH1*	Esophageal (KYSE 150, KYSE 510), Prostate (PC-3), Colon (HT-29)	Fang et al. (2007)
		*SCUBE 2*	Breast (MCF-7, MDA-MB-231)	Sheng et al. (2019)
Genistein	DNMT inhibitor, Promoter demethylation	*EPHB2, GSTP1, BRCA1*	Prostate (DU-145, PC-3)	Adjakly et al. (2011)
		*TP53, PTEN, CDH1, DAPK1, FHIT, RUNX3, SOCS1*	Cervix (HeLa)	Sundaram et al. (2019)
Luteolin	DNMT inhibitor, Promoter demethylation	*P16INK4a*	Colorectal (BE)	Krifa et al. (2014)
		*Nrf2*	Colorectal (HCT116)	Zuo et al. (2018)
		*Nrf2, p53*	Colon (HT-29, SNU-407)	Kang et al. (2019)
Lycopene	DNMT inhibitor, Promoter demethylation	*GSTP1*	Prostate (PC3)	Fu et al. (2014)
Phenethyl isothiocyanate (PEITC)	DNMT inhibitor, Promoter demethylation	*RASSF1A*	Prostate (LNCaP)	Boyanapalli et al. (2016)
		*GSTP1*		Wang et al. (2007)
Quercetin	DNMT inhibitor, Promoter demethylation	*P16INK4a, Er-beta, RASSF1A*	Bladder (EJ, J28, T24)	Ma et al. (2006)
		*DAPK1, BCL2L11, BAX, BNIP3, BNIP3L, APAF1*	Leukemia (HL60, U937)	Alvarez et al. (2018)
		*APC, CDH1, CDH13, DAPK1, FHIT, GSTP1, MGMT, MLH1, PTEN, RARB, RASSF1, SOC51, TIMP3, VHL*	Cervix (HeLa)	Kedhari Sundaram et al. (2019)
Resveratrol	DNMT inhibitor Promoter demethylation	*CRABP2*	Thyroid (THJ-11T, UW228-2)	Liu et al. (2019b)
Rosmarinic acid	DNMT inhibitor	*-*	Breast (MCF7)	Paluszczak et al. (2010)
Shikonin	DNMT inhibitor	*-*	Breast (MCF-7), Cervix (HeLa)	Jang et al. (2015)
Sulforaphane	DNMT inhibitor, Promoter demethylation	*-*	Prostate (PC-3, LNCaP)	Wong et al. (2014)
		*Nrf2*	Colon (Caco-2)	Zhou et al. (2019a)
Triptolide	Promoter demethylation	*WIF-1*	Lung (A549, H460)	Nardi et al. (2018)
Withaferin A	CpG hypermethylation	*ADAM8, PLAU, TNFSF12, ME3, GSTM1*	Breast (MCF-7, MDA-MB-231)	vel Szic et al. (2017)
	CpG hypomethylation	*GLRX2, GFPT2, STX11 and VGF*		

### 2.3 Phytocompounds targeting DNA methylation

An increasing list of different phytocompounds targeting DNA methylation at distinct genes in various cancer cell lines has been discovered in the last two decades. This section summarizes the phytocompounds that have the potential to act as DNA methylation inhibitors and activators altering the tumor suppressor gene expression.

Apigenin, a polyphenol, has been shown to reduce the methylation of CpG sites in the *Nrf2* promoter region, which contributed to increased mRNA levels of downstream genes. *Nrf2* codes a transcription factor that regulates antioxidant enzymes. Furthermore, apigenin also decreased DNMT expression in mouse skin cancer cell line (Paredes-Gonzalez et al., 2014).

*Neurog-1* gene plays a critical role in neuronal differentiation. Curcumin-induced treatment reported demethylation of 14 CpG sites of *Neurog-1* promoter and downstream gene reactivation in prostate cancer cell line (Shu et al., 2011). *BRCA1*, a DNA repair gene, critically regulates cell cycle checkpoints and transcription. Triple-negative breast cancer (TNBC) cell lines were treated with curcumin, which caused the upregulation of the TET1 gene that promoted the *BRCA1* promoter’s hypomethylation. Furthermore, it induced upregulation of DNMT3 that resulted in suppressed expression of proto-oncogene *SNCG* (Al-Yousef et al., 2020). Curcumin treatment reactivated *RARβ* by causing promoter hypomethylation in lung cancer cells. The treatment further resulted in increased RARβ protein and mRNA expression. Curcumin treatment also induced downregulation of DNMT3b, resulting in decreased expression of DNMT3b mRNA levels (Jiang et al., 2015).

Epigallocatechin 3-gallate (EGCG), a polyphenol obtained from green tea, is one of the most studied chemo preventive agents. Besides EGCG, epigallocatechin and epicatechin are also among the major constituents of green tea. Several studies have documented numerous medical benefits of EGCG; regulation of cancer cell growth is one of them (Shankar et al., 2007). According to an *in vitro* study, catechol inhibits DNMT activity by directly inhibiting DNMTs or by causing o-methylation of SAM by methyltransferases that result in increased SAM levels. Treatment of esophageal cancer cell lines with EGCG exhibited unmethylation specific bands for tumor suppressor *p16INK4a*, retinoic acid receptor *β* (*RARβ*), *MGMT*, and DNA repair *hMLH1* genes. Furthermore, it demonstrated that EGCG reactivated the *RARβ* gene in prostate cancer and esophageal cancer cell lines (Fang et al., 2007). In another study, breast cancer cell lines reported downregulation of DNMT expression and restoration of the tumor suppressor gene *SCUBE 2* expression, when treated with EGCG, that resulted in increased E-cadherin expression and suppression of cell migration and invasion (Sheng et al., 2019).

Genistein-induced treatment of HeLa cells reduced promoter 5′CpG methylation levels of various tumor suppressor genes involved in PI3K and MAPK signaling. The restoration of transcription levels of earlier hypermethylated genes (mentioned in Table 2) can be correlated to a decrease in methylation levels (Sundaram et al., 2019). In a triple-negative breast cancer cell, silencing of *BRCA1* is often a result of overexpression of the aryl hydrocarbon receptor. *In vivo* study of the mammary gland of adult mice demonstrated that lifelong treatment with genistein reduced *BRCA1* CpG methylation in the offspring’s mammary tissue (Donovan et al., 2019). Treatment with genistein and daidzein resulted in re-expression of *BRCA1, EPHB2,* and Glutathione S-transferase pi 1 (*GSTP1*) in prostate cancer cells, suggesting a preventive effect of soy phytoestrogens against prostate cancer (Adjakly et al., 2011).

*GSTP1* encodes for a detoxifying enzyme that protects cells from genome-damaging stresses caused by reactive chemical species. Lycopene induced promoter demethylation of *GSTP1* in prostate cancer cells, increasing the mRNA and protein levels. Further treatment with lycopene also decreased the protein levels of DNMT3a. Fu et al. (2014).

The hypermethylated *RASSF1A* promoter in prostate cancer cell line, when treated with phenethyl isothiocyanate (PEITC), reported a decrease in methylation of CpG sites by an average of 90% compared to untreated cells with 98% of methylation at 16 CpG sites. Promoter demethylation of the tumor suppressor gene *RASSF1A* correlated with decreased mRNA expression of DNMT1 and DNMT3A when treated with PEITC (Boyanapalli et al., 2016). PEITC induced CpG demethylation in *GSTP1* promoter of LNCAP cells in a concentration-dependent manner and was almost similar or higher than the commonly used DNMT inhibitor 5-Aza-2′-deoxycytidine. On further analysis by pyrosequencing tool, it was revealed that PEITC treatment significantly reduced CpG methylation at positions 1 and 3 from 89.5% to 73.2% and 61.8%–6.5%, respectively, that was higher than the demethylating activity of 5′-Aza (Wang et al., 2007).

Quercetin inhibited the DNMT activity and resulted in the restoration of various tumor suppressor genes that were earlier hypermethylated at 5′CpG promoter sites in cervical cancer cells (mentioned in Table 2). Furthermore, *in silico* studies revealed that quercetin competitively inhibit DNMTs by binding to its catalytic active sites (Kedhari Sundaram et al., 2019). Quercetin treatment decreases the methylation levels of *P16INK4a, Er-beta,* and *RASSF1A* genes in bladder cancer cell lines. Furthermore, quercetin treatment inhibited the expression of mutant p53 and survivin proteins. P53 maintains cell cycle regulation, DNA repair, and apoptosis, and any alteration in p53 happens to decrease genomic stability and DNA repair. While Survivin acts as antagonists by inhibiting antiapoptotic pathways and promoting mitotic progression (Ma et al., 2006). *In vitro* and *in vivo* studies revealed that quercetin induced demethylation of highly methylated promoter sites of apoptosis-related genes *BCL2L11* and *DAPKlin,* leukemia cell lines (Alvarez et al., 2018).

A study on rodent’s mammary tumor treated with resveratrol indicated a decrease in DNMT 3b expression. The study further reported that 26% of rats developed tumors when treated with a low dose of resveratrol, while only 18% developed tumors with high dose compared to 33% in the control group (Qin et al., 2014). Resveratrol upregulated the expression of cellular retinoic acid binding protein 2 (*CRABP2*) that mediates retinoic acid anticancer pathways in thyroid cancer cell lines by partial demethylation of CpG promoter sites. In addition, resveratrol significantly decreased DNMT1 and DNMT3A expression (Liu X. et al., 2019).

Sulforaphane treatment downregulated DNMT expression and can mediate promoter demethylation in prostate cancer cells (Wong et al., 2014). In colon cancer cells, sulforaphane inhibited the expression of DNMT1 and increased Nrf2 protein expression by decreasing the methylation of Nrf2 promoter region (Zhou J. W. et al., 2019).

In TNBC cells, withaferin A induced hypermethylation of tumor-promoting genes *ADAM8, PLAU, TNFSF12, GSTM1, ME3,* and hypomethylation of *GLRX2, GFPT2, STX11,* and *VGF* (vel Szic et al., 2017).

The reversible epigenetic process of DNA methylation regulates chromosomal integrity, tissue differentiation, and gene expression throughout embryogenesis. However, any aberrant epigenetic modifications during these processes can lead to tumorigenesis. As extensively discussed above, numerous studies provide evidence that these abnormal modifications can be undone by a plethora of phytocompound treatment. Phytocompounds targeting DNA methylation are listed below in Table 2.

## 3 Histone modifications

The eukaryotic DNA is condensed in the form of chromatin. A nucleosome, the basic unit of chromatin, comprises of three parts, i.e., a core nucleosome, linker DNA, and histone H1. Each nucleosome core contains 2 copies of each histone H2A, H2B, H3, and H4, and approximately ∼147bp of DNA wrapped around it, in the form of a histone octamer. Core histone proteins comprise of three structural motifs, i.e., the histone fold regions, their diverse extensions, and histone tails. Histone tails are sites of posttranslational modifications and are extremely basic, which consists mainly of lysine and arginine amino acids (Luger and Richmond, 1998; Kornberg and Lorch, 1999). Histone modifications influence the regulation of chromatin dynamics in processes such as gene regulation, DNA repair, cell proliferation, and apoptosis. In cancer, deregulation of genes involved in these pathways may lead to unwarranted activation of oncogenes or inactivation of tumor suppressor genes (Audia and Campbell, 2016).

### 3.1 Histone acetylation

Histone acetyltransferases (HATs) catalyze the transfer of an acetyl group to the ε-amino group of the lysine side chains utilizing the cofactor acetyl CoA, thereby weakening the DNA and histone interactions. HATs have been classified into two- Type-A and Type-B. Further, HATs can be divided into three major categories- GNAT, MYST, and p300/CBP (Bannister and Kouzarides, 2011). Acetylation at K5 and K12 of newly synthesized H4 histones is catalyzed by type-B HAT (HAT1), along with specific sites at H3 histone (Kouzarides, 2007). Predominant transcriptional repressors, histone deacetylases (HDACs), catalyze the reverse lysine acetylation, thus restoring the positive charge of lysine and stabilizing the chromatin structure. The HDACs have been categorized into four major classes: classes I, II, III, and IV. HDACs 1, 2, 3 and belongs to class I HDACs, while class II comprises of HDACs 4, 5, 6, 7, 9, and 10; and only HDAC11 belongs to class IV HDACs. Sirtuins, another name for class III HDACs, are structurally distinct from the other classes and require a cofactor (NAD^+^) for its activity (Mottet and Castronovo, 2008).

### 3.2 Histone methylation

Lysine and arginine residues are mostly favored for histone methylation. Lysine methyltransferases catalyze the transfer of a methyl group from SAM to a ε-amino group of lysine, whereas arginine methyltransferases catalyze the transfer of a methyl group from SAM to arginine’s ω-amino group. Histone demethylases have the opposite effect to histone methylases, both can activate or repress the transcriptional activity (Bannister et al., 2002). For example, methylation of histone H3 at K9 and K36 may negatively affect the promoter region while a positive one in the coding region (Kouzarides, 2007). Additionally, decreased levels of acetylation of histones H3 and H4 and elevated levels of DNA methyltransferases are usually found in prostate cancer cells (Baumgart and Haendler, 2017).

### 3.3 Histone phosphorylation

Histone phosphorylation is highly versatile and occurs predominantly at serine, threonine, and tyrosine residues. Phosphorylation results from the transfer of a phosphate group from ATP to the hydroxyl group of the amino acid side chain that is catalyzed by histone kinases (Xhemalce et al., 2011).

### 3.4 Histone lactylation

A recent study also revealed that lactate, produced by the incomplete oxidation of glucose through the Warburg effect in cancer cells, can regulate gene expression in macrophages by functioning as a new histone modification, i.e., lactylation. Excessive lactate production, i.e., Warburg effect benefits cancer in number of ways-promotes metastasis, angiogenesis, activation of T cells, polarization of macrophages. Now it is well documented that lactic acid also contributed in epigenetic modifications by adding the lactyl group on the ε-amino group of a lysine residue (Zhang et al., 2019).

In addition, there are diverse sets of posttranslational histone modifications that include deamination, ADP ribosylation, ubiquitylation, and sumoylation but are beyond the scope of this review.

### 3.5 Phytocompounds inhibiting histone modifications

In the last two decades, several dietary compounds have been confirmed to play a substantial role in the reversal of histone onco-modification and a few of the most important of them are discussed in detail below.

In prostate cancer cells, apigenin induced a decrease in HDAC activity, downregulated HDAC1 and 3 expression, and increased acetylation of histones H3 and H4*. In vivo* studies reveled that apigenin treatment reduced tumor growth and a significant decrease in HDAC activity that correlated with increased levels of p21/waf1and Bax protein along with a reduction in protein levels of bcl2 that favored apoptosis in tumor cells of the mice (Pandey et al., 2012). Another *in vivo* study on the breast cancer cell line of athymic nude mice revealed a decrease in HDAC activity on treatment with apigenin in a dose-dependent manner. Acetylation of histone H3 was shown to increase after treatment with apigenin correlated with transcriptional activation of *p21* ^*WAF1/CIP1*^ gene (Tseng et al., 2017). Apigenin treatment lowered the protein expression levels of HDAC1, 3, 4, 5, 6, and 8 in the mouse skin cancer cell line in a dose-dependent manner (Paredes-Gonzalez et al., 2014).

Curcumin treatment resulted in inhibition of cell proliferation in Raji cells of B-NHL cancer. The protein expression levels of HDAC1, 3, and 8 were also found to be downregulated and acetylation of H4 histone increased after treatment with curcumin in a dose- and time-dependent manner (Liu et al., 2005). In HeLa nuclear extracts, curcumin decreases the HDAC activity. Docking studies suggested curcumin as a potent inhibitor of HDAC8 than its carboxylic acid-derived pharmacological counterparts (Bora-Tatar et al., 2009). In human hepatoma Hep3B cells, curcumin induced a decrease in H3 and H4 histone acetylation. Further *in vitro* studies showed curcumin treatment decreased core histone acetylation catalyzed by HAT extracted from Hep3B cancer cell line that suggested the role of HAT in curcumin-induced histone hypoacetylation (Kang et al., 2005).

In a recent study, demethylzeylasteral treatment promoted decrease in tumor progression in liver stem cells by inhibiting H3 lactylation (H3K9la and H3K56la) (Pan et al., 2022).

Green tea polyphenol (GTP) treatment decreased a maximum of 43% HDAC activity in a time-dependent manner in prostate cancer cells, which was similar to Trichostatin A (TSA) that caused 45% inhibition in 24 h. Furthermore, a decrease in protein expression of HDAC1 and 3 was also observed. GTP treatment decreased the mRNA levels of HDAC1, 2, and 3 in a gradual time course, whereas no such changes were observed with TSA. Exposure to GTP further resulted in a 22-fold and 2.2-fold increase in H3 and H4 acetylation, respectively, in a gradual-time course (Pandey et al., 2010). EGCG treatment significantly reduced HDAC activity in skin cancer cell line A431. Acetylation and methylation levels of H3K9 were found to be increased and decreased, respectively, when treated with EGCG. Furthermore, acetylation of H3K4 and H4K5, 12, and 16 were shown to be increased after EGCG treatment, thus reactivating tumor suppressor genes (Nandakumar et al., 2011). In prostate cancer cells, GTP and its major constituent EGCG induced treatment caused a substantial decrease in the expression and activity of HDAC 1, 2, 3, and 8. On further investigation, EGCG acetylated the *p53* gene at K373 and K382, which was found to be diminished when the EGCG treatment was withdrawn after a certain time period (Thakur et al., 2012).

Genistein treatment increased acetylation at H3, H4, and H3 dimethylated at K4 near the transcription start sites of tumor suppressor genes *p21* and *p16* in prostate cancer cell lines. ChIP analysis revealed an elevation in HAT activity, suggesting an increase in transcription level and gene activation after treatment with genistein (Majid et al., 2008). Genistein, eqoul, and AglyMax induced ER-mediated core histone acetylation via modulating the activity of HATs, and daidzein stimulated Erβ mediated histone acetylation (Hong et al., 2004).

Triple-negative breast cancer is considered the most aggressive subtype of breast cancer. An *in vitro* study on the effect of indole-3-carbinol on HCC70 triple-negative breast cancer cell lines has shown to inhibit the overall HDAC activity (Nouriemamzaden et al., 2020).

PEITC treatment of LNCaP prostate cancer cell line significantly reduced the protein levels of HDAC1, 2, 4, and 6 that correlated with promoter demethylation and activation of a tumor suppressor gene, *RASSF1A* (Boyanapalli et al., 2016). Histone hypoacetylation due to excessive HDAC activity is one of the hallmarks of leukemia. Mononuclear extracts from the bone marrow of acute myeloid leukemia patients showed limited or no H3 and H4 histone acetylation. However, after treatment with phenylhexyl isothiocyanate (PHI), there was a significant elevation in H3 and H4 histone acetylation compared to the control cultures (Xiao et al., 2010).

In HL-60 leukemia cancer cell lines, quercetin treatment induced FasL expression that triggered extrinsic apoptotic pathway, protein activation and conformational changes, and activation of ERK and JNK signaling pathways. Furthermore, H3 acetylation was found to be increased in quercetin treated HL-60 cells, and upregulated HAT activity and downregulated HDAC activity together resulted in stimulation of FasL expression (Lee et al., 2011). Leukemia cancer cell lines when treated with quercetin showed an increase in global histone acetylation of H3 and H4 histones. Promoter regions of proapoptotic genes experienced a three-to ten-fold increase in H3 and H4 acetylation in HL-60 and U937 cancer cells. *In vitro* and *in vivo* studies of two human xenograft myeloid leukemia models exhibited a decrease in HDAC1 and 2 activity after treatment with quercetin (Alvarez et al., 2018). Quercetin treatment significantly reduced the HDAC activity in a dose-dependent manner and HMT activity at H3 histone, which may methylate and trimethylate the ninth lysine residue in HeLa cancer cell lines. Molecular docking results of quercetin reported a decline in HDAC activity, suggesting that quercetin can competitively inhibit HDAC2, HDAC4, HDAC7, and HDAC8 by binding to the catalytic residue sites (Kedhari Sundaram et al., 2019).

In breast cancer cell lines, resveratrol decreases the protein levels of arginine methyltransferase PRMT5, lysine methyltransferase EZH2, and lysine deacetylase KDAC in a dose- and time-dependent manner that correlated with an increase in protein levels of *BRAC1, p21,* and *p53* genes. After treatment with resveratrol, near to the proximity of the transcription start site of the mentioned genes, the levels of H4R3me2s and H3K27me3 were found to be decreased while that of H3K9ac and H3K27ac increased (Chatterjee et al., 2019). In human carcinoma cell line, resveratrol treatment increase the protein levels of acH3K9, acH3K14, acH3K12, acH4K5, and acH4K16, suggesting anti-tumor effects of resveratrol (Dai et al., 2020).

In human malignant melanoma cells, sulforaphane, decreases the protein expression levels of HDAC1, 2, 4, and 6. Sulforaphane also decreases the total HDAC activity and protein expression levels of CBP, CBP/p300, and PCAF. Furthermore, the protein expression levels of acH3K9, 14, and 27, and acH3K8 and 12 were significantly reduced after treatment with sulforaphane. The study also revealed a decrease HMT activity of SET7/9, further affecting the methylation at K9, 36, and 79 when treated with sulforaphane (Mitsiogianni et al., 2020). TERT (Telomerase Reverse Transcriptase) is associated with processes such as cell proliferation, senescence, cell differentiation, *etc.*, and any alteration in it contributes to immortality and carcinogenesis. Sulforaphane treatment suppressed HDAC activity in prostate cancer cells and induced an increase in pan-acetylation of H3 and H4 histones of the hTERT promoter (Abbas et al., 2015).

Furthermore, the histone modulation activity of various phytocompounds like diallyl disulfide, garcinol, ginsenoside Rh2, phenyl hexyl isothiocyanate, rosmarinic acid, *etc.*, in distinct cancer types is mentioned in Table 3.

**TABLE 3:** List of phytocompounds modulating histone modifications.

Dietary phytocompound	Mechanism	Genes targeted	Cancer type (cancer cell line)	References
Allicin	H4 acetylation↑	-	Mouse erythroleukemia (DS19)	Link et al. (2013)
Allyl isothiocyanate	H4 acetylation↑	-	Mouse erythroleukemia (DS19)	Lea et al. (2001)
Apigenin	HDAC activity↓	*-*	Prostate (PC-3, 22Rv)	Pandey et al. (2012)
	HDAC expression↓	*p21/waf1*	Breast (MDA-MB-231)	Tseng et al. (2017)
	H3 and H4 acetylation↑			
Curcumin	HDAC activity↓	*-*	Cervix (HeLa)	Bora-Tatar et al. (2009)
	HDAC expression↓		Hepatoma (Hep3B)	Kang et al. (2005)
	H4 acetylation↑		Lymphoma (Raji)	Liu et al. (2005)
	H3 and H4 acetylation↓			
Diallyl disulfide	HDAC activity↓	*p21(waf1/cip1)*	Colon (Caco-2, HT-29)	Druesne et al. (2004)
	H3 and H4 acetylation↑			
Demethylzeylasteral	H3 lactylation↓	*Cyclin D, CDK2, Cyclin E*	Hepatoma (Hep3B, HCCLM3)	Pan et al. (2022)
EGCG	HDAC activity↓	*GSTP1*	Prostate (LNCaP)	Pandey et al. (2010)
	HDAC expression↓	*p16INK4a, Cip1/p21, p53*	Skin (A431)	Nandakumar et al. (2011)
	H3 and H4 acetylation↑			
	H3 methylation↓			
Garcinol	HAT expression ↓	*-*	Esophageal (KYSE150, KYSE450)	Wang et al. (2020)
Genistein	HAT activity↑	*p21WAF1/CIP1, p16INK4a*	Prostate (LNCaP, DuPro, RWPE)	Majid et al. (2008)
	H3 and H4 acetylation↑			
	H3 methylation↑			
Ginsenoside Rh2	HDAC activity↑	*MMP3*	Hepatocellular (HepG2)	Shi et al. (2014)
Indole-3-carbinol	HDAC activity↓	*-*	Breast (HCC70)	Nouriemamzaden et al. (2020)
Kaempferol	HDAC activity↓	*-*	Hepatocellular (HepG2, Hep3B) and Colorectal (HCT-116)	Berger et al. (2013)
Luteolin	HDAC activity ↓	*-*	Colorectal (HCT-116)	Zuo et al. (2018)
Parthenolide	HDAC activity ↓	*-*	Colorectal (HCT-116)	Dawood et al. (2019)
Phenethyl isothiocyanate (PEITC)	HDAC activity↓	*RASSF1A*	Prostate (LNCaP)	Boyanapalli et al. (2016)
Phenylhexyl isothiocyanate	H3 and H4 acetylation↑	*-*	Leukemia (mononuclear extract)	Xiao et al. (2010)
Quercetin	HAT activity↑	*DAPK1, BCL2L11, BAX, APAF1, BNIP3, BNIP3L*	Leukemia (HL-60)	Alvarez et al. (2018)
	HDAC activity↓	*-*	Cervix (HeLa)	Kedhari Sundaram et al. (2019)
	H3and H4 acetylation↑			
	HMT activity↓			
Resveratrol	HMT activity↓	*BRCA1, p53, p21*	Breast (MCF-7, MDA-MB-231)	Chatterjee et al. (2019)
	HMT expression↓	*-*	Renal (ACHN)	Dai et al. (2020)
	HDAC activity↓			
	H3 and H4 acetylation↑			
Rosmarinic acid	HDAC expression↓	*p53, Bax, Bcl-2, PARP-1*	Prostate (PC-3, DU145)	Jang et al. (2018)
Sulforaphane	HMT activity↓	*hTERT*	Prostate (LNCaP)	Abbas et al. (2015)
	HDAC activity↓	*-*	Melanoma (A375)	Mitsiogianni et al. (2020)
	HAT activity↓			
	H3 and H4 acetylation↓			
Thymoquinone	HDAC activity↓	*p21, Maspin, Bax and Bcl-2*	Breast (MCF-7)	Parbin et al. (2016)
Triptolide	HMT activity↓	*-*	Lung (A549, H460)	Nardi et al. (2018)

Based on the studies mentioned above, it is evident that a wide range of phytocompounds (apigenin, curcumin, sulforaphane, resveratrol, genistein, quercetin, *etc.*) possess the ability to target major histone modifications that regulate gene expression of apoptosis, cell proliferation and inflammatory pathways (extensively discussed in section 6), all of which, when dysregulated can lead to carcinogenesis.

## 4 MicroRNAs

Non-coding RNAs without protein or peptide-coding potential are classified into two major categories: long ncRNA (200 nucleotides long) and short ncRNA that consists of miRNAs, siRNA, piwiRNA, *etc.* Non-coding RNAs, initially assumed to be junk in the transcriptome, have now been discovered to play a crucial role in cellular signaling pathways, including those that regulate cancer initiation and progression (Setoyama et al., 2011). miRNAs are single-stranded and 18–20 nucleotides in length that are formed after undergoing complex maturation steps (Figure 3). With the assistance of Drosha (RNase III enzyme) and Pasha (ds-RNA binding endonuclease), the primary miRNA stem-loop structure undergoes numerous modifications, which leaves a 70 nucleotide long pre-miRNA. This pre-miRNA is then transported to the cytoplasm from the nucleus and is subjected to further processing with DICER and TRBP (transactivating response RNA-binding protein) that generates a 22-nucleotide extended miRNA duplex. The duplex associates with the RISC (RNA inducing silencing complex) complex that targets the mRNA for gene regulation (Zhang et al., 2007; Shruti et al., 2011). Several studies have suggested that miRNA can influence cell signaling, regulation, proliferation, and apoptosis by controlling oncogenes and tumor suppressor gene expression (Mishra et al., 2016).

### 4.1 Epi-miRNA

Any alterations during the biogenesis of miRNA or mutation of the factors can have profound implications. A category of miRNAs, termed as epi-miRNAs (epi-miRs), has recently been discovered to modulate the expression of genes encoding epigenetic reader proteins (Dai et al., 2014). Aberrant modulation of epi-miRs can induce epigenetic silencing of tumor suppressor genes or activation of oncogenes, resulting in carcinogenesis. MicroRNAs have been classically categorized as two distinct epi-miRs, namely, OncomiRs and tumor-suppressor miRs that play distinctive roles in tumorigenesis. OncomiRs are generally upregulated, resulting in enhanced cancer cell proliferation and metastasis, whereas tumor-suppressor miRs are downregulated leading to carcinogenesis (Svoronos et al., 2016; Sadakierska-Chudy, 2020). Phytocompounds that are known to alter the epi-miRNAs is the entire effect of the each phytocomponents is supported by a single publication are listed in Table 4.

**TABLE 4:** List of phytocompounds modulating MicroRNA.

Dietary phytocompound	Mechanism	Genes targeted	Cancer type (cancer cell line)	References
Apigenin	miR-16↑	*MMP-9*	Glioma (U87)	Chen et al. (2016)
Celastrol	miR-17–92a↓	*ATG7*	Prostate (LNCaP)	Guo et al. (2016)
Cucurbitacin B	miR- 146-5p↑	*-*	Pancreas (BxPC‐3, MiaPaCa‐2, HPAC, ASPC‐1)	Zhou et al. (2019b)
Curcumin	miR-99a↑	*-*	Retinoblastoma (SO-Rb50, Y-79)	Li et al. (2018)
	miR-34a↑, let-7b↑, miR-200a↑	*Axl, Slug, CD24, Rho-A*	Breast (MDA-MB-231, MCF-10F)	Gallardo et al. (2020)
3,3′- Diindolylmethane	miR-30e↓	*ATG5, LC3*	Gastric (BGC-823, SGC-7901)	Ye et al. (2016)
Ellagitannins	let-7a↓, let-7c↓, let-7d↓, let-7e↑, miR-370↑, miR-373*↑, miR-526b↑	*-*	Hepatocarcinoma (HepG_2_)	Wen et al. (2009)
EGCG	miR-18a↓, miR-34b↓, miR-193b↓, miR-222↓ miR-342↓, miR-16↑, miR-221↑, let-7b↑	*Bcl-2*	Hepatocarcinoma (HepG2)	Tsang and Kwok (2010)
Genistein	miR-574-3p↑	*-*	Prostate (PC3, DU145)	Chiyomaru et al. (2013)
	miR-145↑	*ABCE1*	Retinoblastoma (Y79)	Wei et al. (2017)
Glabridin	miR-148a↑	*-*	Breast (MDA-MB-231, Hs-578T)	Mu et al. (2017)
Gossypol	miR-15a↑	*Bcl-2*	Pituitary (GH3, MMQ)	Tang et al. (2015)
Icariin	miR-625-3p↓	*-*	Thyroid (SW579, TPC1)	Fang et al. (2019)
Kaempferol	miR-340↑	*-*	Lung (A549)	Han et al. (2018)
Lycopene	miR-let-7f-1↑	*-*	Prostate (PC3)	Li et al. (2016)
Methyl jasmonate	miR-101↑	*-*	Colorectal (SW670)	Peng and Zhang (2017)
PEITC	miR-194↑	*-*	Prostate (LNCaP)	Zhang et al. (2016)
Piceatannol	miR-21↓	*PTEN*	Osteosarcoma (MG-63, Saos-2) Colorectal (HCT-116, HT29)	Zheng and Wu (2020)
	miR-129↑	*-*		Zhang et al. (2014)
Quercetin	miR-16↑	*HOXA 10*	Oral (HSC-16, SCC-9)	Zhao et al. (2019)
	miR-146a↑	*-*	Breast (MCF-7, MDA-MB-231)	Tao et al. (2015)
Resveratrol	miR-200c↑	*-*	Colorectal (HCT-116)	Karimi Dermani et al. (2017)
				
Rosmarinic acid	miR-506↑	*-*	Pancreas (Panc-1, SW 1990)	Han et al. (2019)
Sulforaphane	miR-135b-3p↑	*RASAL2*	Pancreas (BxPC-3, PANC-1, AsPC-1)	Yin et al. (2019)
	miR-21↓	*-*	Colorectal (RKO)	Martin et al. (2017)
Thymoquinone	miR-16↑, miR-375↑	*BCL-2, Caspase-3*	Hepatocellular carcinoma (HepG2, Huh7)	Bashir et al. (2020)
Triptolide	miR-193b-3p↑	*KLF4*	Nephroblastoma (G-401, WiT49)	Hang et al. (2019)
Ursolic acid	miR-21↓	*-*	Glioblastoma (U251)	Wang et al. (2012)
Withaferin A	miR-let-7c-5p↑	*CCND1, c-MYC*	Breast (MCF-7)	Prajapati et al. (2022)

### 4.2 Phytocompounds modulating the epi-miRNAs expression

A significant proportion of studies have demonstrated the potential role of phytocompounds in the regulation of epi-miRs in carcinogenesis. For instance, the apigenin-treated glioma cancer cell line U87 exhibited increased miR-16 expression, decreased BCL2 protein expression, and decreased expression of the *MMP-9* gene. Anti-miR-16 transfection inhibited apigenin-induced miR-16 gene expression. Furthermore, anti-miR-16 transfection inhibited apigenin-induced miR-16 gene expression, increased BCL2 protein expression and NF-κB/MMP-9 levels (Chen et al., 2016).

Curcumin induced upregulation of miR-99a in retinoblastoma cancer cell line, SO-Rb50, and Y-79. When the cells were transfected with miR-99a inhibitor, the miR-99a expression decreased that correlated with anti-tumor activity of curcumin through enhancement of miR-99a expression. Phosphorylation levels of JAK1, STAT1, and STAT3 were significantly reduced when treated with curcumin, although no such effect was observed when miR-99a was knocked down (Li et al., 2018). Curcumin-treated breast cancer cell line MCF-10F resulted in decreased gene transcript and protein levels of *Axl, Slug, CD24, and Rho-A*, which are associated with epithelial-mesenchymal transition (EMT). An increased expression of miR-200a, let-7b, and miR-34a was found in the MCF-10F cancer cell line, while only miR-34a expression increased in the MDA-MB-231 cancer cell line after exposure to curcumin. An increase in expression of the examined genes occurred after the knockdown of miR-34a, while the genes were found downregulated when treated with curcumin and transfected with anti-miR-34a. The invasive and migrating capabilities of MCF-10F decreased when cells were transfected with anti-miR-34a and treated with curcumin (Gallardo et al., 2020).

Ellagitannin treated hepatocarcinoma cells showed a decrease in cell proliferation. The treatment further downregulated and upregulated the expression of various mi-RNAs (Table 4) (Wen et al., 2009).

Hepatocarcinoma cell line HepG2 exhibited decreased growth when treated with EGCG in a dose-dependent manner. miR-18a, miR-34b, miR-193b, miR-222, and miR-342 were found to be downregulated, while miR-16, miR-221, and let-7b were found to be upregulated after treatment with EGCG. *Bcl-2* expression was suppressed after transfection with miR-16. Furthermore, transfection with miR-16 enhanced the activity of EGCG in *Bcl-2* suppression and induction of apoptosis (Tsang and Kwok, 2010). Nasopharyngeal cancer cell line CNE2 was treated with EGCG where 32 miRNAs exhibited > 2-fold changes that have been shown to modulate cancer development, out of which 29 miRNAs were found to be upregulated and 1 miRNA was downregulated in a dose-dependent manner (Li et al., 2017).

Genistein-treated prostate cancer cell lines, exhibited an increase in miR-574-3p expression compared to the control. Transfection with pre-miR-574-3p miRNA precursors into PCa cell lines led to a significant increase in miR-574-3p expression and decreased cell invasion. In an *in vivo* study, the transfection of DU145 cells with miR-574-3p subcutaneously into nude mice resulted in tumor suppression due to overexpression of miR-574-3p (Chiyomaru et al., 2013). A significant increase in miR-145 expression was observed in genistein-treated retinoblastoma cancer cell line (Y79). Genistein treatment also reduced cancer cell proliferation and induced apoptosis. Y79 cells transfected with miR-145 specific siRNA resulted in the restoration of colony formation capacity and suppression of cell apoptosis that was induced due to genistein treatment. In silico studies suggested *ABCE1* gene to be a potential target of miR-145. Furthermore, an *in vivo* study on the xenograft nude mice model revealed the suppression of tumor growth in Y79 cells administered with genistein (Wei et al., 2017).

Treatment with lycopene induced upregulation of miR-let-7f-1 in a dose and time-dependent manner in prostate cancer cells. Furthermore, transfection with miR mimics led to inhibition of cell proliferation and apoptosis induction (Li et al., 2016).

PEITC treated prostate cancer cell lines exhibited an increase in the expression of miR-194. Furthermore, the expression of the two matrix metalloproteinase, *MMP2 and MMP9*, which significantly contributes to tumor progression in terms of migration, invasion, and metastasis, decreased when treated with PEITC. An *in silico* study revealed *BMP1* is a potential target of miR-194 and its inhibition tends to downregulate MMP2 and MMP9 levels (Zhang et al., 2016).

Quercetin-treated oral cancer cells exhibited an increase in miR-16 expression. Cell viability, migration, and invasive capabilities of oral cancer cells were found to be repressed when transfected with miR-16. HomeboxA10 (*HOXA10*) was found to be targeted by miR-16 after the bioinformatics analysis of the binding sites of HOXA10 and miR-16. *HOXA10* is generally involved in the proliferation, invasion, and migration of cancer cells and is one of the potential biomarkers in oral cancer. Overexpressed miR-16 downregulated the protein levels of *HOXA10*, while the opposite was observed in miR-16 knockdown cells (Zhao et al., 2019). In breast cancer cells, miR-146a expression increased after treatment with quercetin in a dose-dependent manner. Furthermore, growth of the cells was inhibited after transfection with miR-146a mimic and treatment with quercetin. A substantial elevation in the expression of *Bax* and cleaved caspases was observed when transfected with miR-146a. Quercetin treatment for 8 weeks increased miR-146a expression and reduced tumor growth in a nude mouse orthotopic xenograft model (Tao et al., 2015).

Colorectal cancer cell line HCT-116 exhibited a decrease in cell viability in a dose-and-time-dependent manner when treated with resveratrol. HCT-116 cells transfected with LNA miR inhibitor showed a dramatic decline in miR-200c expression compared with the un-transfected and scrambled groups. After treatment with resveratrol, miR-200c expression increased significantly in both transfected and un-transfected cells. Resveratrol treatment reduced the mRNA and protein expression of vimentin and ZEB1, while that of E-cadherin increased in both groups that correlated with EMT induction (Karimi Dermani et al., 2017).

Rosmarinic acid decreased cell viability, cell proliferation, invasion, and migration and suppressed EMT while promoting apoptosis in pancreatic cancer cell lines. Further treatment resulted in increased miR-506 levels that correlated with suppression of MMP2 and MMP16 proteins. The xenograft mouse model also exhibited a reduction in tumor growth after treatment with rosmarinic acid (Han et al., 2019).

Sulforaphane-treated pancreatic cancer cell lines showed a substantial increase in miR135b-5p. Cells transfected with liposomes of miR-135b-5p mimics exhibited overexpression of miR-135b-3p that resulted in reduced cell viability, migration, and colony-forming capacity. *In silico* analysis revealed *RASAL2* to be a potential target of miR-135b-3p. Furthermore, an *in vivo* studies on tumor xenografts, where BxPC-3 cells were transfected with miR-135b-3p mimics, resulted in decreased tumor size that correlated with overexpression of miR-135b-3p and *RASAL2* (Yin et al., 2019). In colorectal cancer cell, sulforaphane treatment inhibited oncogenic miR-21, decreased cell viability, induced apoptosis, and downregulated the expression of *hTERT* (Martin et al., 2017).

In human glioblastoma cells, miR-21 levels were increased that has been shown to target a positive regulator of apoptosis, the *PDCD4* gene. Ursolic acid treatment decreased cell proliferation and induced apoptosis while suppressing the levels of miR-21 that eventually led to enhanced expression of *PDCD4* (Wang et al., 2012).

All these studies discuss the therapeutic effect of phytocompounds targeting epi-miRs that either decreases or increases miRNA level to eventually reduce tumorigenesis. An elaborate list of phytocompounds modulating miRNA in specific cancer cell types is provided in Table 4.

## 5 Plant-based compounds as epigenetic modulator of genes involved in apoptosis, autophagy, inflammation and oxidative stress

As discussed above, aberrant epigenetic modulation can alter the function and activity of typical genes and transcriptional factors involved in various essential pathways like autophagy, apoptosis inflammation, *etc.*, that can lead to cancer.

### 5.1 Autophagy

Autophagy plays a dual role in cancer by promoting tumorigenesis or suppressing tumor progression. To satisfy the high metabolic requirements of proliferating tumor cells, autophagy recycles the cells’ intracellular constituents to supply nutrients, while suppression or inhibition of autophagy genes may result in cancer cell death. Autophagy also promotes inflammation in tumor cells, which eventually results in tumor progression (Yun and Lee, 2018). Post-translational modifications like acetylation, phosphorylation, ubiquitination, nitrosylation can influence autophagy by regulating the activity of ATG proteins and the expression of genes involved in autophagy (Botti-Millet et al., 2016). As described above, many studies have shown that deregulation of ATG genes through different epigenetic modulations can cause tumor progression. For instance, curcumin treatment inhibited DNMT1 and DNMT3B expression in prostate cancer cells that resulted in promoter hypomethylation of miR-143 and miR-145. This restoration of miRNAs further downregulated *ATG2B* expression, thus inhibiting autophagy (Liu et al., 2017).

### 5.2 Apoptosis

Dysregulation of apoptosis is one of the hallmarks of cancer. Cancer cells tend to survive longer and accumulate mutations due to deregulation of apoptosis over a period of time. Furthermore, deviations from normal apoptotic pathways can enhance the invasiveness of cancer cells and promote angiogenesis. In most cancer cells, BCL-2 is generally overexpressed while the function of caspases is found to be disabled (Pfeffer and Singh, 2018). As mentioned above, emerging studies have shown that phytocompounds can correct epigenetic modulations that interfere with apoptotic pathways that result in cancer progression.

### 5.3 Inflammation and oxidative stress

Excessive aggregation of reactive oxygen species (ROS) has been observed in cancer. ROS is involved in inflammation, cell transformation, tumor cell survival, proliferation, invasion, angiogenesis, and metastasis, mediated through transcription factors such as NF-κB, AP-1, STAT3, *etc.* (Gupta et al., 2012; Prasad et al., 2017). Furthermore, the overexpression of MMPs can be correlated with enhanced invasiveness and angiogenesis in distinct cancer types (Reunanen and Kähäri, 2013). Besides this, ROS can also regulate the expression of multiple tumor suppressor genes like *p53, Nrf2, PTEN, and Rb* (Gupta et al., 2012). Nrf2 plays a crucial role in homeostasis maintenance and regulation of genes that produce anti-inflammatory and anti-cancer effects (Wu et al., 2019). Additionally, it is evident from the studies mentioned that abnormal epigenetic modulation can alter the function and activity of transcription factors and genes involved in the production of ROS in cancer. However, distinct phytocompounds (curcumin, resveratrol, berberine, luteolin, *etc.*) as mentioned above, possess the ability to reverse the activity of all these transcription factors and genes involved in the accumulation of ROS back to normal, which have been implicated in abnormal epigenetic modulation.

Therefore, it is evident from all these studies and observations that autophagy, apoptosis, inflammation, and oxidative stress play a crucial role in cancer. This warrants additional studies to understand the underlying mechanisms of genes, transcription factors, and pathways implicated in cancer. Furthermore, these implications can also be used as biomarkers in the diagnosis of distinct cancer types.

## 6 Toxic effects of phytocompounds

Most *in vitro* and *in vivo* studies suggested the use of high concentrations of phytocompounds for tumor growth suppression and chemoprevention. However, in humans, certain elevated levels of these phytocompounds may not be reached due to their poor bioavailability and may lack therapeutic efficacy. Studies also show that such high doses of phytocompounds over a prolonged duration may exhibit high toxicity. For instance, resveratrol was administered to rats for 4 weeks in a dose-dependent manner. Serious side effects such as increased kidney weight, increased plasma BUN, and creatinine levels, which gradually contributed to nephrotoxicity, were observed at a concentration of 3,000 mg resveratrol per kg body weight of rats (Crowell et al., 2004). In another study on rat thymocytes, resveratrol at 10 µM concentration raised the shrunken cell population and exerted cytotoxic effects on normal cells by inducing apoptosis (Fujimoto et al., 2009). Despite evidence indicating the antioxidant activity of curcumin, several studies also demonstrated the pro-oxidant activity of curcumin, increasing ROS levels in cells (Yoshino et al., 2004; Su et al., 2006). The pro-oxidant nature of curcumin would increase cellular ROS levels at higher doses, potentially contributing to carcinogenesis (López-Lázaro, 2008). Furthermore, excessive quercetin intake exacerbates tumorigenicity induced by a chemical carcinogen N-ethyl-N′-nitro-N-nitrosoguanidine (ENNG), in the duodenum of mice (Matsukawa et al., 2002). While the immense health-promoting benefits of epigallocatechin-3-gallate, a study demonstrated that high dose administration of EGCG in mice resulted in hepatotoxicity correlated with inhibition of antioxidant enzymes and Nrf2 targeted genes (Wang et al., 2015). Treatment of female CD-1 mice with genistein at environmentally appropriate doses resulted in irregular estrous cycles, early reproductive senescence, impaired ovarian activity, diminished fertility, while at higher doses, the number of stillbirths increased (Jefferson et al., 2005). In an *in vivo* study on Swiss mice, administration of higher doses (100 and 200 mg/kg) of apigenin resulted in elevated serum levels of alanine aminotransferase, aspartate aminotransferase, alkaline phosphatase, and reactive oxygen species that eventually contributed to liver damage (Singh et al., 2012).

In addition to this, a variety of phytocompounds that humans have consumed for decades have been shown to possess carcinogenic properties. For example, capsaicin, cycasin, phytoestrogens, safrole, amygdalin, phorbol esters, pyrrolizidine alkaloids, obtained from different dietary sources, may serve as potential carcinogens or promoters of tumors (Bode and Dong, 2015; Guldiken et al., 2018).

Several experiments have been performed with numerous phytocompounds to examine their possible positive and detrimental biological effects. However, all natural substances should not be considered healthy and attention should also be given to their toxic dose-related effects. Furthermore, all these observations warrant additional studies and humanized clinical trials regarding the adverse and chronic effects of high toxic doses of distinct phytocompounds.

## 7 Clinical trials

Phytocompounds disrupt the process of epigenetic transformation in cancer by directly inhibiting epigenetic modulations and also by modulating epigenetic regulators. As more evidence emerges highlighting the therapeutic importance of epigenetic modifications of cancer and the ability of phytocompounds to target these modifications further endorses their clinical relevance. The potential of phytocompounds already known to have anticancer effects to target epigenetics should be thoroughly assessed and may be used as a criterion for inclusion of compounds for further clinical evaluation. For this context, we review a few plant-based compounds known to suppress epigenetic modifications that are currently in various phases of clinical studies below.

Several preclinical studies have demonstrated the efficacy of phytocompounds in regulating epigenetic changes for chemotherapeutic purposes, there have been insufficient clinical trials to back this up. For instance, curcumin regulated the activity of MMP-2, Bcl-2, Nrf-2, Bax, and PIK3/AkT signaling in a randomized clinical trial to study the effects of paclitaxel and curcumin combined in breast cancer to mitigate multidrug resistance. In patients with advanced breast cancer, curcumin is under investigation as monotherapy (NCT03980509) or in combination with paclitaxel in phase II clinical trial (NCT03072992). The main object of these clinical studies is to determine the effect of curcumin on the development of advanced breast cancer and to estimate the risk of adverse effects. In a phase II study of the effect of sulforaphane-rich extracts in men with recurrent cancer, 20 subjects were treated with 200 µmoles/day of sulforaphane extract for 20 weeks. Out of the 20 subjects, six showed an increase in histone acetylation following sulforaphane treatment (Alumkal et al., 2015). In an ongoing randomized pilot study in phase I clinical trial investigating the impact of quercetin on EGCG uptake in prostate cancer, the downregulating effects of quercetin on enzyme function and protein and gene expression of COMT (catechol-O-methyltransferase) and DNMT1 are being assessed (NCT01912820). Treatment with 3,3′-Diindolylmethane enhanced the expression of let-7, miR-27b, miR-34a, miR-124, miR-200, and miR-320, which led to downregulation of androgen receptor activity, EMT, and stem cell markers, all of which were associated with enzalutamide resistance in Castration-resistant prostate cancer (Li and Sarkar, 2016).

Additionally, these natural compounds can be categorized in to 3 different phases as per their success in treating cancer are compounds under preclinical trials, clinical trials and those are used in current cancer therapy. Phytocompounds those are in pre-clinical trials are ursolic acid (Prasad et al., 2012; Zhang et al., 2018), withaferin A (Choi and Kim, 2015; Suman et al., 2016; Kuppusamy et al., 2017), curcumin (Kunnumakkara et al., 2017), baicalein (Dou et al., 2018; Tao et al., 2018), EGCG (Thangapazham et al., 2007), apigenin (Chang et al., 2018; Yan et al., 2018), genistein (Zhang et al., 2013; Hsiao et al., 2019), resveratrol (Banerjee et al., 2002), sulphorane (Qazi et al., 2010), thymoquinone (Zhu et al., 2016), *etc.* Phytocompounds that go for clinical trial focus on three major aspects of cancer research: 1) improving the response of cancer cells towards standard chemo- and radiotherapy, 2) reducing the severe adverse effects of standard cancer therapy, and 3) looking for unwanted interactions with standard therapy. Preclinical studies have shown the effectiveness of various phytochemicals as mentioned above. The phytochemicals which are currently under clinical trials against various cancers are Berberine (NCT03281096), curcumin (NCT03072992), EGCG (NCT02891538), lycopene (NCT03167268), quercetin (NCT01912820), resveratrol (NCT01476592) and sulphorane (NCT03232138). Several compounds are in clinical use are vincristine, vinblastine, paclitaxel, etoposide to name a few. This underscores the significance of natural chemicals and the imperative to further investigate their potential in developing the most efficacious and secure pharmaceutical interventions for cancer therapy.

Research on cancer-fighting phytocompounds is still in their infancy since only a small number of phytocompounds (such as paclitaxel, docetaxel, and vinblastine) have been granted clinical use licenses. With an increasing understanding of the range of anticancer effects of plant-based compounds, such as inhibition of cancer epigenetics, there is an urgent need to screen more therapeutically effective phytocompounds. In addition, no clinical investigation has been conducted to date to assess how phytocompounds modify micro RNAs in the context of cancer epigenetics therapy. In the majority of related clinical studies, methodological flaws such as the absence of a control or placebo group, small sample sizes, and brief trial duration are observed. Therefore, for many phytochemicals, it is too early to conclude their anticancer actions and hence large-scale and well-controlled clinical trials are required to validate their efficacies, adverse effects, and safeties before their use for the treatment of cancer. To achieve the international standard, promising phytochemicals require extensive standardization in terms of methods for evaluating their bioavailability, efficacy, safety, quality, composition, manufacturing processes, regulatory and approval practices. In order to enhance the clinical assessment of phytocompounds, we must establish an evaluation pipeline. A methodology that takes into account drug optimization, effectiveness assessment, tissue toxicity and distribution, chemical accessibility, pharmacokinetics, absorption, and most importantly bioavailability should be created for an enhanced evaluation of phytocompounds. The stability and availability of phytocompounds in blood can be improved by using stable synthetic analogues, chemically modified derivatives, micelle-coated medications, liposomal conjugates, phospholipid complexes, adjuvants, and nanoparticles. The effectiveness of plant-based medicines can also be augmented by using other techniques including structure-activity relationship, directed optimization, and pharmacophore-oriented molecular design (Xiao et al., 2016; Atanasov et al., 2021; Mohammadi et al., 2022).

## 8 Conclusion and future prospects

Epigenetic aberrations remarkably contribute to cancer incidence. As mentioned above, distinct studies highlighted the potential of phytocompounds in preventing tumorigenesis through regulation of epigenetic modulation by targeting the activity and expression of DNMTs, HDACs, HMTs, epi-miRNAs. The dietary phytocompounds analyzed tend to modulate epigenetic modifications *in vitro* and in some *in vivo* cancer models, thus inhibiting or suppressing cancer cell viability, proliferation, and growth. However, the limited number of studies and insufficient preclinical and clinical data on the effect of phytocompounds on the epigenetic landscape still remains a challenge. Future research should focus on clinical studies regarding the optimal dose and duration of phytocompounds as epidrugs. One of the studies exhibited that a low dietary dose of resveratrol compared with a 200-fold higher dose tends to suppress colorectal cancer development in human and mice tissues (Cai et al., 2015). This result indicates that a low dose of phytocompounds can inhibit tumor progression, thus making it essential to analyze the optimal dose and toxicity of the phytocompounds. To develop anticancer therapeutics, the issue of poor bioavailability of phytocompounds needs to be addressed. Furthermore, significant epidemiological studies revealed that phytocompounds interact with other bioactive compounds that may interfere with their intestinal absorption (Phan et al., 2018). A promising approach to overcome these challenges is the utilization of modern drug delivery systems such as nanoparticles, micelles, liposomes, *etc.*, to enhance bioavailability and overcome systemic toxicity (Aqil et al., 2013). Accumulating shreds of evidence have also demonstrated the synergistic effects of various phytochemicals with chemotherapeutic drugs to be more effective in chemoprevention and cure (Tan and Norhaizan, 2019). However, research regarding the mechanism and course of action of these combined phytocompounds and drugs is still in their infancy. More studies are essential to fully comprehend the mode of action of phytocompounds that have been shown to target a single cell type or are tissue/organ-specific. Besides *in vivo* and *in vitro* studies, mechanistic studies using bioinformatics and high-throughput sequencing methods can help us to better understand and target altered epigenetic modifications in cancer. Dietary phytocompounds (as listed in Tables 2–4) may offer a cost-effective method for chemoprevention, hence improving global health by decreasing the incidence of cancer. Numerous shreds of evidence point to the potential of phytocompounds to target aberrant epigenetic alterations in different forms of cancer. As was already established, genistein functions as a DNMT inhibitor, promotes histone acetylation, and increases levels of miRNAs, all of which contribute to therapeutic impact on prostate cancer. Such phytochemicals need to undergo a thorough evaluation for preclinical and clinical investigations taking into consideration their therapeutic potential in the treatment of cancer. Our study concludes by highlighting the potential of natural compounds in addressing the epigenetic vulnerabilities of cancer, as well as the possible therapeutic benefits that can be identified by advancing our knowledge in this field.

## Figures and Tables

**Table:** 

**ABCE1**	ATP-binding cassette subfamily E, member 1
**ADAM8**	ADAM Metallopeptidase Domain 8
**ATG5**	Autophagy related 5
**BAX**	BCL2 Associated X
**BCL2L11**	Bcl-2-like protein 11
**BMP1**	Bone Morphogenetic Protein 1
**BNIP3**	BCL2 Interacting Protein 3
**BRCA-1**	Breast Cancer gene 1
**CCND1**	Cyclin D1
**CDH1**	Cadherin-1
**CRABP2**	Cellular retinoic acid-binding protein 2
**DAPK1**	Death-Associated Protein Kinase 1
**DNMT**	DNA methyl transferases
**EGCG**	Epigallocatechin-3-gallate
**EPHB2**	Ephrin type-B receptor 2
**FHIT**	Fragile Histidine Triad Diadenosine Triphosphatase
**GFPT2**	glutamine-fructose-6-phosphate transaminase 2
**GLRX2**	glutaredoxin 2
**GNAT**	Gcn5-related N-acetyltransferase
**GSTM1**	glutathione S-transferase mu 1
**GSTP1**	Glutathione S-transferase pi 1
**HAT**	Histone acetyltransferases
**HDAC**	Histone deacetylases
**hMLH1**	mutL homolog 1
**HMT**	Histone methyltransferases
**JAK1**	Janus Kinase1
**MAPK**	mitogen-activated protein kinase
**ME3**	malic enzyme 3
**MGMT**	O-6-Methylguanine-DNA Methyltransferase
**MMP**	Matrix metalloproteinases
**Neurog-1**	Neurogenin 1
**Nrf2**	nuclear factor erythroid 2–related factor 2
**PDCD4**	Programmed cell death protein 4
**PI3K**	Phosphatidylinositol-3-kinase
**PLAU**	Plasminogen Activator, Urokinase
**PTEN**	Phosphatase and tensin homolog
**RARβ**	retinoic acid receptor beta
**RASSF1A**	Ras association domain family 1 isoform A
**Rho-A**	ras homolog family member A
**RISC**	RNA-induced silencing complex
**RUNX3**	runt-related transcription factor 3
**SAM**	S-adenosylmethionine synthase
**SCUBE 2**	Signal Peptide, CUB Domain And EGF Like Domain Containing 2
**SNCG**	Synuclein Gamma
**SOCS1**	Suppressor of Cytokine Signaling 1
**STAT1**	Signal transducer and activator of transcription 1
**STX11**	syntaxin 11
**TET1**	Ten-eleven translocation methylcytosine dioxygenase 1
**TNBC**	Triple-negative breast cancer
**TNFSF12**	Tumor necrosis factor ligand superfamily member 12
**TRBP**	transactivation response element RNA-binding protein
**TERT**	Telomerase Reverse Transcriptase
**VGF**	VGF nerve growth factor inducible
